# On Muscæ Volitantes

**Published:** 1829-07-01

**Authors:** 


### XVII.

### ROYAL WESTMINSTER OPHTHALMIC HOSPITAL.

### MUSCÆ VOLITANTES.

Mr. Guthrie said muscæ volitantes were of two kinds; one, unaccompanied by defective vision, the other attended by very impaired sight. The first kind were very common. The patient complains "of a variety of appearances moving before the eye, such as small threads or filaments, assuming the form of worms, zigzags, or spots of greater or less dimensions, but generally small; little globules or webs, or luminous spots sometimes surrounded by a halo, which always move before the eye, and are never fixed.

"These are most readily seen on raising the eye quickly from the ground towards the sky, when they appear to ascend whilst the eye is in motion, and to descend on its becoming fixed steadily upwards, as if they had been disturbed from, and were returning to their original situation below the axis of vision. Of the different kinds of muscæ volitantes, the filamentous particles, turning and twisting in various directions, are the most common; two or three of which are generally more conspicuous than the rest, although accompanied by an infinity of others less distinguishable, intermingled with small globules, which fall like a fine mist when the eye has been gently raised and fixed on a white wall, or on the sky on a clear day. The filamentous particles, being apparently the lightest, descend the last, assuming the appearance of twisted semi-transparent tubes, or worms, spotted in different places.

"In the evening, or by candle-light, these spots are scarcely to be observed; they are not very perceptible in a room which is rather dark; are but imperfectly seen when looking at the flame of a candle, and but feebly marked when the eyes are raised to the sky, with the lids shut, on a fine clear day. They appear much more brilliant on a clear or bright day, when the lids are half closed; they are also very distinct on a misty day, or when attention is paid to them in light reflected from water or snow. These spots always appear to sink below the axis of vision by their own weight, when the eye is simply turned upwards; and this opinion seems to obtain great support from their falling and collecting, as it were, into a focus in the axis of vision, when this point is made the most dependent by bending the head forward and looking on a white sandy soil. In this way the patient can readily examine them; or by lying down in the open air and looking at the sky, with the head and eyes turned back, they will be found to ascend and lodge themselves in what is the upper, but, in that position, the most dependent part of the eye. The principal diagnostic mark of these appearances is their mobility, which distinguishes them in a very decided manner from the fixed spots often perceived in the eye, and which depend on opacity of the lens, or a defective state of the retina.

"Muscæ volitantes are incurable; seldom pass a certain point; and when the patient is assured of their not proceeding further, appear to be lost sight of, and to give no inconvenience, unless when the attention is directed to them. They are very rarely followed by cataract or amaurosis, and it is a great consolation to the patient to be assured that they are not dangerous."

Of the second kind Mr. Guthrie said, a well marked case had been related in the March Number of the Medico-Chirurgical Review, as extracted from the Revue Médicale. "The patient complained of defective sight, and on examining the eye a number of small bodies were perceived floating about in the posterior chamber of the eye, glittering with a kind of phosphoric brilliancy." He had seen two cases of the kind; one of a poor man at the hospital, some years ago, who came but once. The sight was very defective, and on dilating the pupil with the belladonna, a number of shining spots were seen floating about, as the eye moved, evidently deeply seated in the vitreous humor, yet connected with each other. The second case is that of a distinguished General Officer, in whose right eye, for three years past, a similar appearance was observable, until the last few months, when cataract supervened. He has had very defective sight with that eye for years, so as only to be able to distinguish a person without ascertaining his features at the usual distance at which he distinctly recognized him with the other. On dilating the pupil, a fine membrane or web was seen floating deep in the posterior part of the eye, rising or falling with its motion. The whole of the web could be distinguished, looking like a fine veil, spotted in places with shining spots, and which did not alter their appearance in whatever light they were examined. In describing them to the patient, Mr. Guthrie said, he had compared them to the stars in the American flag, when floating in the wind. The supervention of cataract has prevented their being seen, whilst it distinctly marks their situation, and has induced him to form an unfavourable prognosis, indeed to decline recommending an operation on that eye. The patient, however, Mr. Guthrie said, had received a more favourable opinion on this point, and if he submitted to an operation, as he was recommended to do, he would not fail to make known the result, in order that a correct opinion might be formed in these cases for the future. The distinction between the two kinds of disease, he considered to be, that in the first, the liquor Morgagni was its seat. In the second, the hyaloid membrane lining the septa of the vitreous humor was detached and floating in it.

---

